# Metabolic Bone Diseases—A Topic of Great Diversity

**DOI:** 10.3390/jcm11216447

**Published:** 2022-10-31

**Authors:** Heinrich Resch, Afrodite Zendeli, Roland Kocijan

**Affiliations:** Faculty of Medicine, Sigmund Freud Universität Vienna, 1020 Wien, Austria

The progress in research has improved the understanding of the epidemiology and pathogenesis of osteoporosis and bone disorders in general. Metabolic bone changes have advanced beyond the simple models of explanation of age-related loss in bone mass, combined with simple and easy assessment of fracture risk by the FRAX model. This was also shown in a Spanish retrospective population-based cohort study on patients suffering from a fragility fracture. The incidence rate of index fragility fractures and obtained information on the subsequent fractures and death during a follow-up of up to three years were assessed [1]. Beyond fracture risk assessment and bone mineral density (BMD) measurements using DEXA, invasive and noninvasive methods for the assessment of trabecular and cortical bone structure, strength and material properties have been developed. Bone microstructural deterioration at the distal radial and the unfractured distal tibia was quantified in a small but well-defined homogeneous group using high-resolution peripheral quantitative computed tomography [2]. It was shown that stress fractures were associated with compromised cortical and trabecular microstructure, changes which are not covered by standard BMD diagnostics.

In healthy people, peak bone mass and bone remodeling are stable for many years unless a secondary, stimulative cause of bone loss is present. In women, the decline in estrogen as early as in perimenopause results in an imbalance of bone remodeling, such that resorptive processes exceed formative processes and, as a consequence, bone mass decreases. Premature ovarian insufficiency as an example of hypergonadotropic hypogonadism caused by impaired ovarian function before the age of 40 is associated with an increased risk of BMD loss and development of osteopenia and osteoporosis which poses an important problem for public health [3]. At the same time, with these remodeling characteristics, a deterioration of bone architecture and disturbances in skeletal integrity occur. Thus, early initiation of full-dose hormone replacement therapy (HRT) has a significant and positive influence on bone mass in these patients.

Knowledge of bone relates primarily to primary osteoporosis in which bone loss can be attributed to aging per se or the known hormonal consequences of aging. A large number of heterogeneous causes (e.g., metabolic, inflammatory, autoimmune, vascular, renal diseases, genetic disorders and even drugs), defined as secondary causes of osteoporosis, may induce bone loss or structural deterioration through different mechanisms [4,5,6].

In a study on Gaucher disease (GD), standard biomarkers such as TRAP5b levels showed a positive correlation with GD biomarkers, including plasma glucosylsphingosine (lyso-Gb1) and macrophage activation markers, CCL18 as an example [7].

Secondary osteoporosis refers to those conditions that reflect adverse consequences of the primary disease itself which is not bone-related at first sight or caused by side-effects of pharmacotherapies being the standard treatment in those diseases. In this context, diabetic patients with carcinoma in situ under metformin therapy presented lower osteoporosis rates than those who were not receiving metformin therapy [8].

Although these secondary causes of osteoporosis are the most frequently observed causes of unexpected bone loss, they can only be diagnosed by a high degree of suspicion and clinical experience, performing the appropriate investigations. In inflammatory disorders such as rheumatoid arthritis or chronic inflammatory bowel diseases, but also vascular diseases, T-cell activation and consequently pro-inflammatory cascades trigger the increased expression of T-cell-derived RANKL [9,10]. In addition, there is a new biomarker signature of bone-related miRNAs which is promising in certain clinical features [11]. Glucocorticoids, often used to control disease activity, decrease the osteoblasts in number and function and additionally inhibit OPG expression. The ubiquitous occurrence of disease-related secondary changes in bone metabolism implies that numerous medical disciplines need to interact. Especially if surgical interventions and surgical fracture repair are necessary, initiation of specific osteoporosis medication such as bisphosphonates can avoid refractures after surgery [12,13].

Age-related bone loss occurs also in men; however, the mode of this process is still not as well-investigated, leading to an insufficient understanding of the development of the disease in comparison to females. Age-related declines in testosterone are important determinants of bone loss in males which can be reverted due to testosterone replacement [14].

The accelerated progress in bone research has resulted in the development of pharmacologic approaches to minimize or even reverse further bone loss in circumstances where changes in calcium and bone metabolism derive from kidney failure or transplanting the central organ in calcium regulation and homeostasis. In this context, denosumab is able to increase BMD in those patients [15].

Since bone remodeling depends on interactions between formative and resorptive processes, it makes sense to determine the most effective pharmacotherapy as well as the right timepoint to initiate treatment in the concept of sequential therapy [16,17,18]. Denosumab is already approved as an effective drug to reduce fracture risk with seemingly fewer adverse events than have been reported for other monoclonal antibodies used to treat diseases other than osteoporosis. Understanding the mechanisms by which sclerostin as a central regulator of bone formation can be manipulated by an antibody to permit excessive and fast formation of new bone, and molecules that interact with Wnt signaling and LRP5 are linked will finally lead to the development of other therapeutic drugs [19,20,21,22,23].

While this is highly exciting and represents significant progress in maintaining the integrity of bone, it remains to be seen whether this will lead to medications more effective in reducing fracture risk than those drugs currently available.

Screening for secondary causes of osteoporosis and the search for new modes of action should present a substantial part of osteoporosis management. With this Special Issue, we hope to encourage discussion of the current management of osteoporosis and related metabolic bone diseases.
